# Analysis of the impact of emergency care on the incidence of in-hospital complications in patients with acute abdomen and the incidence of complications

**DOI:** 10.3389/fpubh.2025.1612625

**Published:** 2025-06-19

**Authors:** Ruimian Jin, Yuquan Deng, Jing Yang

**Affiliations:** Emergency Department, Longhua District People's Hospital, Shenzhen, China

**Keywords:** grading emergency nursing intervention, acute abdomen, incidence of complications, patient satisfaction, graded nursing model

## Abstract

**Objective:**

To investigate the effect of graded emergency nursing intervention on the incidence of in-hospital complications in patients with acute abdomen and to evaluate patient satisfaction with nursing care.

**Methods:**

Between June 2021 and June 2023, 100 patients with acute abdomen (85 with acute appendicitis) were randomly assigned to a control group (routine nursing care, *n* = 50) or an emergency care (EC) group (graded emergency intervention, *n* = 50). Graded emergency nursing intervention was implemented based on the Emergency Severity Index (ESI) version 4, which stratifies patients from Level I (life-threatening) to Level V (non-urgent). The EC group received structured emergency triage by trained nursing teams, including systematic protocols for patient observation, inquiry, physical examination, and condition analysis. Outcomes included emergency care efficiency indicators (consultation time, examination time, emergency department stay, trauma control time, hospital stay), complication rates (e.g., abdominal infection, hemorrhage, puncture site infection, subcutaneous emphysema), clinical symptom recovery (abdominal pain duration, gastrointestinal recovery time), and nursing satisfaction scores. Data were analyzed using t-tests and chi-square tests via SPSS 21.0, with significance set at *p* < 0.05.

**Results:**

The EC group showed significantly lower rates of in-hospital complications (2% vs. 14%, *p* < 0.05), faster clinical response times (shorter consultation and examination times, reduced emergency department and hospital stay durations), and quicker symptom recovery compared to the control group (*p* < 0.05). Nursing satisfaction scores were also significantly higher in the EC group (*p* < 0.05).

**Conclusion:**

Graded emergency nursing intervention—based on triage acuity, structured symptom assessment, and trained response teams—effectively reduces the incidence of complications, enhances emergency response efficiency, shortens recovery and hospital stay durations, and improves patient satisfaction. This approach is clinically valuable and recommended for broader implementation.

## Introduction

Due to societal developments and shifts in lifestyle, the incidence of acute abdomen is steadily increasing each year, making it a common emergency medical issue (1). Acute abdomen, a surgical urgency characterized by heterogeneous etiologies, demonstrates distinct epidemiological patterns: non-specific abdominal pain constitutes the most frequent presentation (24–44.3% across study cohorts), followed by acute appendicitis (15.9–28.1%), acute biliary pathologies (2.9–9.7%), and age-dependent entities such as bowel obstruction or diverticulitis in older adult populations. Notably, acute appendicitis emerges as the predominant indication for surgical intervention, accounting for two-thirds of pediatric acute abdomen cases requiring operative management (2). Failing to address acute abdomen promptly can elevate the risk of complications, including infection, high fever, and shock, further jeopardizing the patient’s life (3). Patients typically exhibit gastrointestinal symptoms like abdominal pain and bloating, along with systemic reactions such as fever and jaundice. Without timely standard treatment, the condition can progress, leading to various complications, including life-threatening situations (4).

Managing acute abdomen can be challenging due to its complex causes, involvement of multiple medical specialties, and varying clinical manifestations. Currently, the diagnosis of acute abdomen primarily relies on clinical evaluation through comprehensive history-taking and physical examination. The combined use of abdominal ultrasound and computed tomography (CT) is generally reserved for diagnostically ambiguous cases or suspected surgical emergencies requiring anatomical clarification, rather than being routinely employed as first-line diagnostic tools (5). During the assessment, patients cannot provide accurate and timely answers about the disease state, which makes it difficult to diagnose it, thereby prolonging the treatment time and missing the best treatment options (6). Therefore, to prevent delays and ensure timely intervention, it’s essential to complement medical care with scientifically grounded nursing interventions, fostering collaboration with healthcare professionals for systematic rescue efforts and effective disease management (7–9).

Grading emergency nursing intervention encompasses a wide range of responsibilities, including pre-hospital care, patient transfers, in-hospital treatment, monitoring, and ongoing nursing care. Given the unique challenges of the emergency department, characterized by a high patient influx and the complexity of their medical conditions, it becomes crucial for triage nurses to make precise assessments of patients (10). The graded nursing model prioritizes the accurate assessment of patients and employs a systematic approach to scientific triage based on individual patient conditions. It also implements targeted interventions to ensure a streamlined care process (11). In recent times, both local and international scholars have applied the principles of the graded emergency nursing model to the management of critical conditions like acute pancreatitis, acute chest pain, acute abdomen, and others. This approach involves making well-considered care plans tailored to patients’ specific conditions, which leads to a reduction in waiting times. Furthermore, it enables critically ill patients to receive effective treatment during the critical “golden window” time period, ultimately enhancing the quality of care (10, 12).

The objective of this research was to introduce the concept of graded grading emergency nursing intervention and assess its impact on the management of patients with acute abdomen conditions, as well as to gage patient satisfaction with nursing care. The outcomes of this study will establish a theoretical foundation for the future development of grading emergency nursing intervention.

## Patients and methods

### Patient information

Between June 2021 and June 2023, a total of 100 patients with acute abdomen (85 cases of acute appendicitis) were enrolled. The randomization process strictly followed the CONSORT 2010 guideline (13). An independent statistician generated the random allocation sequence via SPSS 28.0 software, implementing allocation concealment through sequentially numbered, sealed opaque envelopes. Patients were divided into control (*n* = 50) and emergency care (EC) groups (*n* = 50) based on enrollment order (1–100). Envelopes were opened post-enrollment to prevent selection bias. Following random number generation and ascending sorting, the first 50 patients were allocated to the EC group. Blinding was not feasible for patients/nursing staff due to distinct protocols, but outcome assessors and data analysts remained blinded. Demographics: control group (30 males/20 females), EC group (27 males/23 females).

Inclusion criteria:

Aged 17–64 years with acute abdomen diagnosis (acute appendicitis).

Exclusion criteria:

Craniocerebral trauma.

Chronic systemic diseases (cardiovascular, respiratory, renal, hepatic).

Mental illness or intellectual disability.

### Nursing intervention methods

In this study, the intervention period for both the control and EC groups was defined as the duration from patient admission to the emergency department until transfer out of the emergency unit and subsequent hospital discharge.

Patients in the control group received standard care protocols, which included: upon receiving a call at the emergency center, the relevant emergency personnel were immediately organized to transport the patient to the emergency department. The emergency team was composed of team leaders and experienced rescue personnel. Routine nursing procedures were followed, and nursing satisfaction questionnaire surveys were conducted.

Patients in the EC group received emergency care based on the following components.

#### Triage system

Triage followed the Emergency Severity Index (ESI), version 4 (13), where the initial condition of the patient was assessed and categorized into five levels:

Level I (Immediate): Life-threatening conditions requiring resuscitation (e.g., hemodynamic instability).

Level II (Emergent): Critical conditions requiring evaluation within 10 min (e.g., severe abdominal pain with fever).

Level III (Urgent): Stable but requiring evaluation within 30 min (e.g., moderate pain with localized tenderness).

Level IV (Less Urgent): Non-urgent cases assessed hourly (e.g., mild pain without systemic symptoms).

Level V (Non-Urgent): Referred to outpatient care (e.g., chronic abdominal discomfort).

#### Personnel training

A scaled nursing team was established, consisting of senior nurses and internal hospital staff. Team members underwent theoretical and practical training, including joint examinations focused on emergency classification methods. Patient satisfaction surveys were administered to evaluate the effectiveness of emergency triage nursing.

#### Specific measures for scale-based interventions

1. Observation: Upon hospital arrival, a detailed assessment was conducted, including evaluation of skin condition, mental and consciousness state, and physical activity. Signs such as pained expressions, consciousness disturbances, restlessness with tense abdominal muscles, or sweating with irritability were noted as indicators of severe or shock-prone conditions.
2. Inquiry: Immediate questioning upon admission was performed to gather detailed information about the chief complaints, symptom characteristics, and mechanisms. In conscious patients, the timing, duration, and persistence of symptoms were recorded. For female patients, menstrual history was obtained. Postprandial abdominal pain raised suspicion of acute pancreatitis; nighttime pain with hematochezia suggested gastrointestinal bleeding lesions.
3. Examination: Vital signs were closely monitored, and abdominal examinations were performed. Abdominal pain with peritoneal irritation suggested perforation or ulcers. A positive Murphy’s sign indicated acute cholecystitis, while McBurney’s point tenderness suggested acute appendicitis. Radiation pain in female patients indicated possible uterine or adnexal pathology.
4. Analysis: A comprehensive analysis and assessment were conducted based on the findings. Patients were categorized appropriately according to severity scales and then transferred to the relevant departments for further care.

### Observation indicators

1. First aid indicators: The two groups were compared in terms of first aid time, consultation time, examination time, emergency stay time, trauma control time, and length of hospital stay.
2. Nursing satisfaction: Patient satisfaction with nursing services was assessed using a hospital-developed nursing satisfaction questionnaire, comprising four domains: nursing staff’s disease knowledge, attitude, treatment timeliness, and operational skills. Each item was rated on a 4-point Likert scale, where 1 indicated dissatisfaction and 5 indicated high satisfaction. Each domain was worth 25 points, resulting in a total score of 100. A higher total score indicated a higher level of satisfaction. The overall satisfaction rate was calculated as: overall satisfaction = number of very satisfied cases + satisfied cases.
3. Clinical symptom recovery and hospitalization time: Recovery indicators included abdominal pain duration and gastrointestinal function recovery time, which were compared between the two groups.
4. Incidence of complications: Complications including subcutaneous emphysema, bleeding, puncture hole infection, and abdominal infection were recorded and compared. The overall complication rate was calculated as follows: overall complication rate = (number of complication cases [subcutaneous emphysema, bleeding, puncture hole infection, and abdominal infection] / total number of cases in each group) × 100%.

### Statistical analysis

All statistical analyses were performed using SPSS version 21.0. Measurement data were expressed as mean ± standard deviation (x ± s) and analyzed using the *t*-test. Categorical data were expressed as percentages (%) and compared using the chi-square (*χ*^2^) test. A *p*-value < 0.05 was considered statistically significant.

## Results

### Comparison of baseline data

The two groups of patients were comparable in terms of baseline data such as gender and age (*p* > 0.05). There were 30 male and 20 female patients in the control group, while the EC group consisted of 27 male and 23 female patients. Regarding the age, the minimum age in the control group was 17, and the maximum was 64. The minimum age in the EC group was 18, and the maximum age was 61 (Table 1).

**Table 1:** Comparison of baseline data between the two groups (x ± s).

Index	Control group	EC group	*t*/*x*^2^	*p*
Cases	50	50	-	-
Gender (Male/Female)	30/20	27/23	0.367	0.545
Minimum age	17	18	-	-
Maximum age	64	61	-	-
Average age	34.42 ± 7.12	34.84 ± 8.71	−0.264	0.792

*EC group, Emergency care group.

### Comparison of first aid-indicators

**Figure 1:**
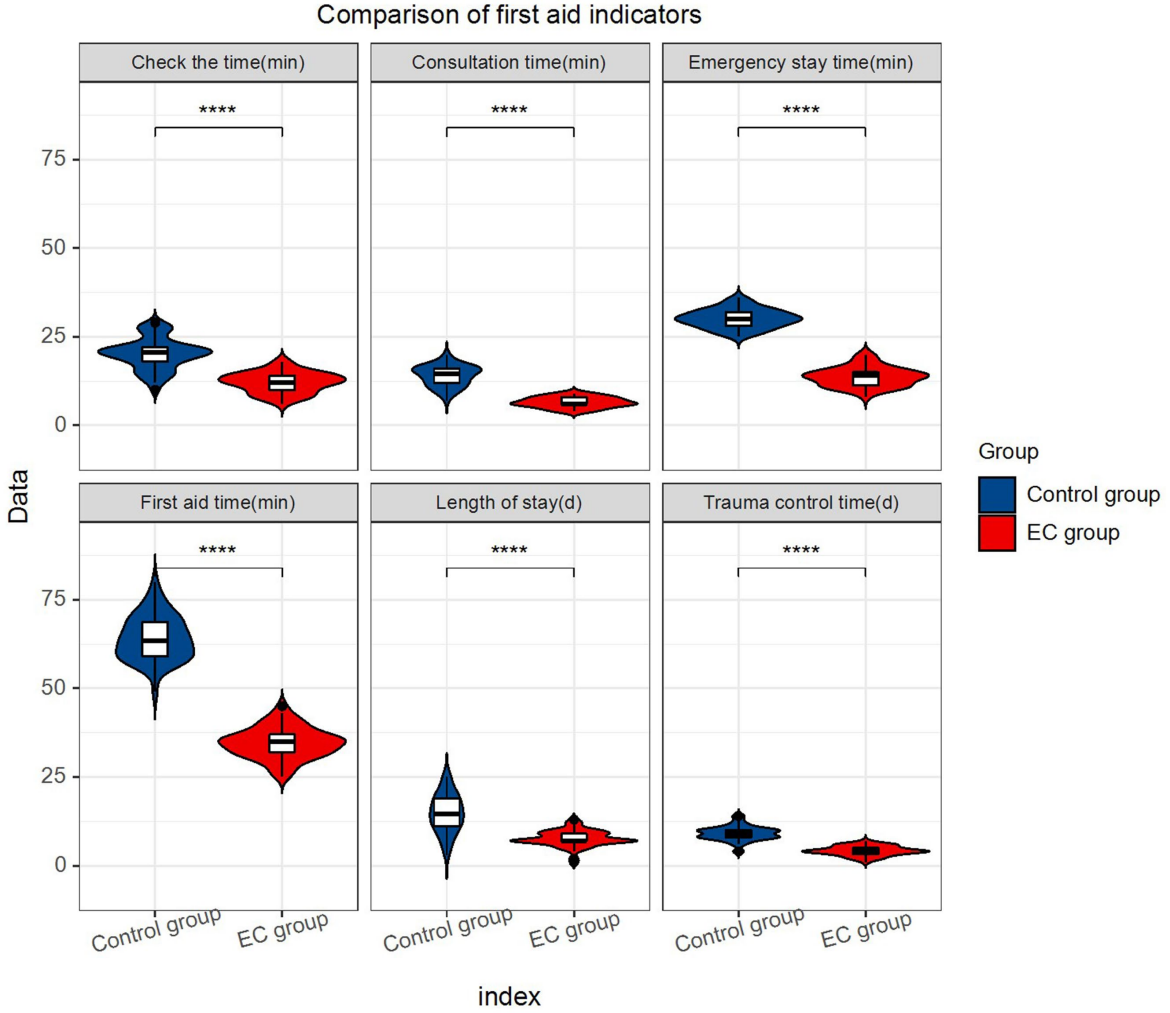
Comparison of first aid indicators.

Analysis of the first aid indicators between the two groups of patients showed that there was a significant difference in the first aid time, consultation time, check-up time, emergency stay time, trauma control time, and length of stay. The EC group had an advantage regarding the first-aid indicators over the control group (*p* < 0.0001) (Table 2; Figure 1).

**Table 2:** Comparison of first aid indicators (x ± s).

Index	Control group (*n* = 50)	EC group (*n* = 50)	*t*/*x*^2^	*p*
First aid time (min)	64.20 ± 6.27	34.62 ± 4.32	27.470	0.000
Consultation time (min)	14.00 ± 2.91	6.52 ± 1.46	16.246	0.000
Check-up time (min)	20.20 ± 4.39	12.16 ± 2.87	10.839	0.000
Emergency stay time (min)	30.16 ± 2.61	13.70 ± 2.72	30.875	0.000
Trauma control time (d)	9.08 ± 2.07	4.18 ± 1.34	14.051	0.000
Length of stay (d)	14.78 ± 5.41	7.64 ± 2.31	8.583	0.000

### Comparison of recovery time from clinical symptoms

**Figure 2:**
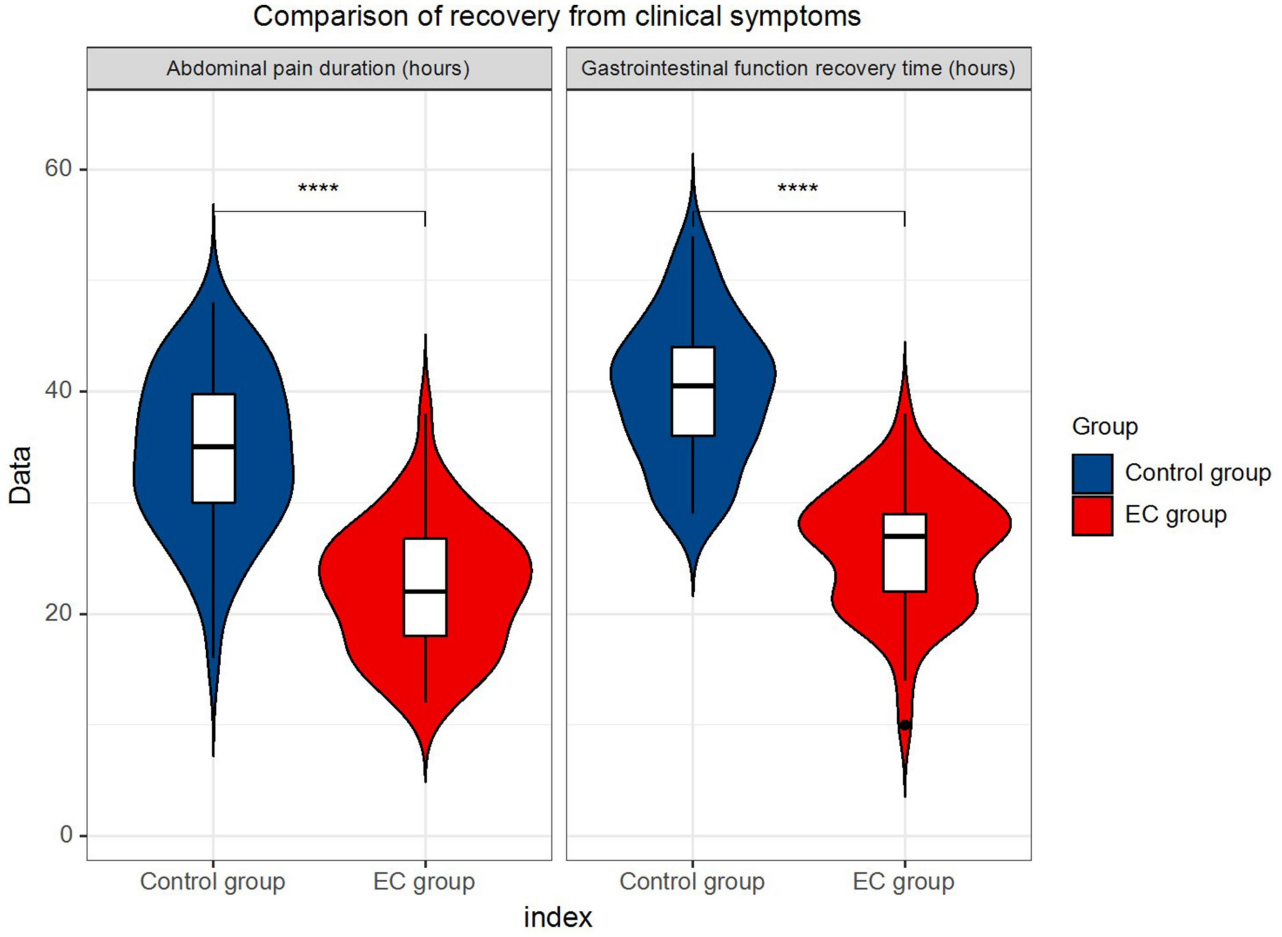
Comparison of recovery from clinical symptoms.

Regarding the comparison of recovery time from the clinical symptoms, the results showed that the EC group was significantly better than the control group in regard to both the duration of abdominal pain and the recovery time of the gastrointestinal function (*p* < 0.0001) (Table 3; Figure 2).

**Table 3:** Comparison of recovery time from clinical symptoms (x ± s).

Group	*n*	Abdominal pain duration (hours)	Gastrointestinal function recovery time (hours)
Control group	50	34.56 ± 7.14	40.06 ± 6.42
EC group	50	22.30 ± 5.76	25.82 ± 5.54
*t*/*x*^2^	-	9.615	12.049
*p*	-	0.000	0.000

### Comparison of incidence of complications

Data demonstrated that the incidence of complications, including subcutaneous emphysema, bleeding, puncture hole infection, and abdominal infection, were lower in the EC group than in the control group (2% vs. 14%), and the difference was statistically significant (*p* < 0.05) (Table 4).

**Table 4:** Comparison of complication rate [*n* (%)].

Group	*n*	Subcutaneous emphysema	Bleeding	Puncture hole infection	Abdominal infection	Overall complication rate
Control group	50	3 (6)	1 (2)	1 (2)	2 (4)	7 (14)
EC group	50	1 (2)	0 (0)	0 (0)	0 (0)	1 (2)
*t*/*x*^2^	-	1.042	1.01	1.01	2.041	5.029
*p*	-	0.307	0.315	0.315	0.153	0.025

### Nursing satisfaction comparison

Evaluation of the patient’s satisfaction with the services provided by the nurses revealed that patients in the EC group were more satisfied with nursing services compared to the patients in the control group (98% vs. 86%), and this difference was statistically significant (*p* < 0.05) (Table 5).

**Table 5:** Comparison of nursing satisfaction [*n* (%)].

Group	*n*	Very satisfied	Satisfied	Less satisfied	Not satisfied	Overall satisfaction rate
Control group	50	31 (62)	8 (16)	4 (8)	7 (14)	43 (86)
EC group	50	40 (80)	7 (14)	2 (4)	1 (2)	49 (98)
*t*/*x*^2^	-	-	-	-	-	4.891
*p*	-	-	-	-	-	0.027

## Discussion

Acute abdomen is a common condition in the emergency department, characterized by acute abdominal pain as the primary clinical symptom. Abdominal pain involves multiple tissues and organs, with a sudden onset, complexity, and rapid changes. It is often accompanied by symptoms such as severe abdominal pain, vomiting, and potential blood loss, as well as complications like shock and electrolyte imbalances (14). Due to the various underlying causes of this condition, treatment methods may vary. Therefore, providing a correct diagnosis and appropriate nursing measures is of utmost importance. Despite significant advancements in medical knowledge and practice, clinical diagnostic errors still occur in approximately 10% of acute abdomen cases, which can impede patient care, lead to poor outcomes, and even result in fatalities (15, 16). This poses significant challenges and burdens for both patients and their families. Hence, the key to reducing postoperative complications and enhancing treatment outcomes lies in delivering accurate and timely treatment and nursing interventions.

In this study, grading emergency nursing intervention was provided to patients in the EC group. This care included reinforcing first aid measures, conducting a comprehensive assessment of the patient’s condition following consultation, and developing targeted and predictive care plans based on clinical manifestations and assessment results (17, 18). Once the patient’s condition was stabilized, precise triage was conducted based on knowledge of diseases, followed by referral to the appropriate department for treatment. This nursing plan was systematic and comprehensive, effectively reducing hospitalization time and improving treatment outcomes. Additionally, given the rising incidence of medical disputes in the emergency department, our department enhanced the management of rescue records to maintain a clear record of the entire nursing and rescue process. This not only served as a basis for patient treatment but also provided a legal foundation when needed, helping to avoid unnecessary complications. As a result of these comprehensive measures, the incidence of complications in the EC group was only 2%, aligning with the findings of Avallin et al. (19). This demonstrates the effectiveness of this nursing method but also places higher demands on the first aid awareness and professional knowledge of emergency nurses. Consequently, nursing staff must strengthen their understanding of first aid, enhance their professional knowledge, become familiar with disease characteristics, promptly and accurately detect issues, and provide swift solutions to ensure effective patient care and improve treatment success rates (20). Furthermore, through appropriate treatment, patients’ hospitalization durations have significantly decreased, leading to savings in medical resources and hospitalization expenses, and a notable increase in patient satisfaction. This is of significant importance in alleviating the often-tense nurse–patient relationship.

Acute appendicitis is a common condition requiring surgical treatment, characterized by rapid onset and severe symptoms that often lead to adverse psychological effects on patients, impacting treatment outcomes (21, 22). During the treatment period, patients may develop various complications that can impact their prognosis. Therefore, it is crucial to help patients rebuild their confidence in treatment and promote postoperative recovery through comprehensive assessment and analysis of their condition. To address this, a comprehensive approach to emergency care encompassing preoperative, intraoperative, and postoperative care is crucial (23, 24). This involves effective communication to alleviate patient fears, provide information, and offer psychological support, as well as preoperative fasting and hydration. Intraoperative care emphasizes gentle surgical techniques to protect intestinal function, while postoperative care focuses on dietary guidance and psychological interventions for improved recovery (25, 26). In this study, when compared to the control group, patients in the EC group demonstrated significant improvements in first aid indicators and other clinical parameters. These findings indicate that comprehensive nursing care significantly enhances the quality of life for hospitalized patients with acute abdomen and accelerates their recovery (27, 28). Furthermore, the study observed a lower incidence of complications in the EC group compared to the control group. Given the wide range of conditions encompassed by acute abdomen and the unclear pathogenesis of acute appendicitis, multiple departments are often involved in treatment. Moreover, patients may vary significantly in their conditions, necessitating a holistic approach to patient care. Therefore, emergency care during treatment can effectively prevent complications (29, 30).

Currently, there is a lack of standardized research or widely accepted protocols specifically addressing the stratification of emergency nursing interventions for patients with acute abdomen. Existing studies in emergency care primarily focus on clinical treatment strategies, surgical timing, or individual nursing measures, with limited discussion on how to systematically grade or classify nursing interventions according to patient condition severity. This study applied a graded emergency nursing intervention model based on clinical assessment, aiming to provide differentiated care strategies tailored to patient needs. The present findings offer data support for this model, suggesting its potential value in clinical settings and indicating the need for further investigation through large-scale, multicenter studies.

The study also revealed that patients in the EC group had higher nursing satisfaction compared to the control group. This underscores the capacity of comprehensive emergency care to alleviate patients’ negative emotions, provide multidimensional care for those with acute abdomen, and significantly improve patient satisfaction. These data emphasize that comprehensive emergency care yields superior outcomes compared to routine care (31, 32).

In emergency care, medical staff should possess specific qualities and knowledge. Firstly, they need to patiently explain the patient’s condition in detail, providing information about the disease and outlining the positive treatment prospects to help patients accept their situation calmly and comprehend effective treatment methods. This approach helps alleviate the negative emotions associated with the illness and contributes to the patient’s recovery process. To excel in these tasks, medical staff must empathize with patients with infectious diseases, understanding the psychological pressures they face, and exhibit patience, compassion, and care in their work. As limitation of our study and in future research, we will increase the sample size to enable more diverse and targeted nursing interventions. Specifically; (1) Implementing psychological interventions by actively listening to patients, aiming to alleviate their fear of surgery; (2) Providing dietary guidance; (3) Conducting relaxation training, such as deep breathing exercises, to promote relaxation; (4) Offering education on disease knowledge, explaining treatment plans, and emphasizing the importance of cooperation during surgery, possibly utilizing visual aids and informational materials; (5) Providing instructions for medication use, ensuring patients take medications correctly and on time; (6) Advising patients to avoid factors that increase intra-abdominal pressure, including strategies to prevent constipation, coughing, hiccups, vomiting, and ensuring they stay warm to prevent cold-induced coughs; (7) Promoting the utilization of medical insurance to reduce the financial burden on patients and their families. Although this study provides important evidence for the clinical effectiveness of tiered emergency nursing interventions, it still has several limitations. First, as a single-center study conducted in a tertiary hospital in Shenzhen, the generalizability of its findings may be limited by regional differences in healthcare levels and institutional treatment protocols. Therefore, future multi-center studies involving populations from diverse regions and socioeconomic backgrounds are needed to validate the universality of the results. Second, although the sample size of 100 cases met the statistical power requirements for the primary outcome (*α* = 0.05, *β* = 0.2), its ability to detect subgroup differences and rare complications (such as subcutaneous emphysema) remains limited. *Post hoc* power analysis indicated that the power to detect the observed reduction in complication rates (14% vs. 2%) was 78%, slightly below the conventional threshold of 80%. Future studies should improve sensitivity and statistical power by conducting prospective sample size calculations.

In summary, the findings of this research suggest that the implementation of graded emergency care can effectively reduce the incidence of complications, improve treatment outcomes, and enhance the nurse–patient relationship. The results obtained from this model demonstrate its value for widespread application, and it is recommended for adoption in clinical practice.

## Data Availability

The original contributions presented in the study are included in the article/supplementary material, further inquiries can be directed to the corresponding author.
